# Long-distance vocalizations of spotted hyenas contain individual, but not group, signatures

**DOI:** 10.1098/rspb.2022.0548

**Published:** 2022-07-27

**Authors:** Kenna D. S. Lehmann, Frants H. Jensen, Andrew S. Gersick, Ariana Strandburg-Peshkin, Kay E. Holekamp

**Affiliations:** ^1^ School of Biological Sciences, University of Nebraska—Lincoln, 1101T Street, Lincoln, NE 68588, USA; ^2^ Department of Biology, Syracuse University, 107 College Place, Syracuse, NY 13244, USA; ^3^ Biology Department, Woods Hole Oceanographic Institution, Woods Hole, MA 02543, USA; ^4^ Dept of Ecology and Evolutionary Biology, Princeton University, 106A Guyot Hall, Princeton, NJ 08544, USA; ^5^ Biology Department, University of Konstanz, Universitätsstrasse 10, 78464 Konstanz, Germany; ^6^ Centre for the Advanced Study of Collective Behaviour, University of Konstanz, Universitätsstrasse 10, 78464 Konstanz, Germany; ^7^ Department for the Ecology of Animal Societies, Max Planck Institute of Animal Behaviour, Bücklestrasse 5a, 78467 Konstanz, Germany; ^8^ Department of Integrative Biology, Michigan State University, 288 Farm Lane, East Lansing, MI 48824 USA; ^9^ Ecology, Evolution, and Behavior Program, Michigan State University, 293 Farm Lane, East Lansing, MI 48824, USA

**Keywords:** animal communication, long-distance signals, individual signatures, group signatures

## Abstract

In animal societies, identity signals are common, mediate interactions within groups, and allow individuals to discriminate group-mates from out-group competitors. However, individual recognition becomes increasingly challenging as group size increases and as signals must be transmitted over greater distances. Group vocal signatures may evolve when successful in-group/out-group distinctions are at the crux of fitness-relevant decisions, but group signatures alone are insufficient when differentiated within-group relationships are important for decision-making. Spotted hyenas are social carnivores that live in stable clans of less than 125 individuals composed of multiple unrelated matrilines. Clan members cooperate to defend resources and communal territories from neighbouring clans and other mega carnivores; this collective defence is mediated by long-range (up to 5 km range) recruitment vocalizations, called whoops. Here, we use machine learning to determine that spotted hyena whoops contain individual but not group signatures, and that fundamental frequency features which propagate well are critical for individual discrimination. For effective clan-level cooperation, hyenas face the cognitive challenge of remembering and recognizing individual voices at long range. We show that serial redundancy in whoop bouts increases individual classification accuracy and thus extended call bouts used by hyenas probably evolved to overcome the challenges of communicating individual identity at long distance.

## 1. Introduction

In complex animal societies, signal receivers face several categorization tasks in addition to detection; to respond adaptively to a signal, they must be able to correctly identify it as relevant or irrelevant to their own interests, and determine whether and how to respond [1]. These categorization tasks become more difficult as social interactions increase in complexity, as social group size increases, as unpredictable variation increases in environmental noise, or as signal transmission is otherwise compromised. These factors make animal communication particularly challenging in fission–fusion societies, where individuals are often widely dispersed. How then do signals evolve to be easily detected and distinguishable enough to transfer relevant information among many individuals in a complex, dispersed social group?

In large groups with differentiated relationships within groups and competition both within and among groups, receivers often need to know the identity or group membership of the caller because it is necessary for receivers to tailor their response to the current situation [2]. When the identity of the signalling individual is important [3–5], signals should emphasize individually distinctive information [6]. For example, signature whistles in bottlenose dolphins are unique to individuals [7] and stable across decades [8], allowing male dolphins to form and maintain complicated multi-level cooperative relationships [9,10]. However, individual recognition becomes increasingly challenging as group size increases because the larger signal set becomes increasingly difficult to discriminate [11,12]. In many species (e.g. wolves [13] chimpanzees [14], green wood hoopoes [15], orca whales [16,17] and sperm whales [18,19]) one or more signals encode information on the group membership of the caller (i.e. ‘group signature’), either in the absence of, or in addition to individual identity information (i.e. ‘individual signature’) [6]. Thus, we expect group vocal signatures to evolve when groups are large and successful in-group/out-group distinctions are at the crux of fitness-relevant decisions, while individual recognition systems may be necessary when relationships *within* groups require further decision-making because relationships vary among group-mates or change quickly over time.

An additional problem arises when individual or group identity information must be transmitted over long distances. In such cases, information is predicted to be encoded in call features that are most robust to sound propagation. Therefore, long-distance acoustic signals should be tonal because pure tones travel better than broadband noise, which is susceptible to scattering [20]. Since loss of energy owing to sound absorption increases with frequency, and because tonal signals are less susceptible to scattering [20], long-range calls tend to be tonal, low-frequency signals. Long-range propagation also leads to increased signal reverberation from reflections and refraction, favouring information encoded in frequency modulations rather than amplitude modulations [20,21]). Distinctive voice features that might allow for recognizing individuals at short range, such as subtle differences in formant spacing shaped by vocal tract filtration [22], are unlikely to be useful for long-range identification of callers.

To maximize detection and improve discrimination, signallers can increase amplitude, avoid noise either in time or signal space, or increase redundancy in a signal (summarized in [1]), but not all these strategies are options for long-distance signallers. Signals that are optimized for long-range transmission often operate near-physiological amplitude limits already, and senders seldom have much control over noise conditions, especially for distant receivers. By contrast, signal redundancy via repetition [23,24] is probably low cost, and this call feature is under behavioural control. Here we inquire whether spotted hyenas (*Crocuta crocuta*) have individual signatures, group signatures, neither, or both in the their long-distance vocalizations.

Spotted hyenas are large carnivores that live in social groups, called ‘clans’, which may contain up to 125 members in the prey-rich plains of eastern Africa [25,26]. There, clan members cooperate to defend a communal territory (13–76 km^2^, [25]) and other critical resources against neighbouring clans and other large carnivores. Each clan contains multiple unrelated matrilines of females and their offspring, as well as one or more immigrant males that sire most young [27]. Female hyenas are philopatric, but most males disperse from their natal clan to join a new clan at 2 to 6 years of age [28–31]. Each hyena clan is structured by a strict linear dominance hierarchy [32] where social rank determines priority of access to resources. Relationships among clan-mates thus vary based on rank, sex, age and kinship. These dynamic relationships are further complicated by the fission–fusion nature of hyena clans [33,34]. Although clan membership is largely stable over time, individuals and sub-groups break apart and come together many times each day at myriad locations within the clan territory [35].

The long-distance call of spotted hyenas, the whoop vocalization, has multiple hypothesized functions [28], including recruitment and coordination of movements by clan-mates within their territory [36,37], sexual advertisement [38], finding specific group-mates [39] and territory maintenance [30,40]. The whoop vocalization is loud and can be heard up to 5 km away [28,41]. It is most often emitted in bouts that range from 2 to 34 whoops [41], and each whoop is a harmonic, frequency-modulated, tonal call. Three whoop types have been described [41] and are not specific to behavioural context [37,38]. At least at short range, mothers recognize and respond strongly to the whoops of their young offspring [39] and individual distinctiveness in cub whoops appears to extend into adulthood [38]. The fundamental frequency of a whoop provides reliable information about the caller's age class and, for adult callers, information about sex as well [37]. Thus, whoops appear to encode information about the caller's age, sex, location, affective state and individual identity.

To effectively defend their key resources and compete against other large carnivores, hyenas rely on long-distance communication to coordinate a large number of clan members dispersed over an expansive territory. Given the large clan size (far beyond typical group size for both wolves and sperm whales) and the need for effective discrimination of clan-mates for cooperative territory defence, we inquire whether, like wolves [13], hyenas have evolved a group-specific label to simplify the cognitive challenge of identifying clan members at long range.

Here, we use machine learning to test whether the hyenas' long-distance vocalizations contain group and/or individual signatures, and to identify call features that can facilitate discrimination. We then use these results to quantify how serial redundancy in extended whoop bouts can affect individual classification accuracy. Finally, we discuss the implications of these findings for understanding signal evolution and acoustic communication in large, socially complex and spatially dispersed species.

## 2. Methods

### (a) Study animals and call recordings

We recorded whoops emitted by spotted hyenas from four clans monitored by the Mara Hyena Project in the Maasai Mara National Reserve, Kenya (electronic supplementary material, figure S1). We identified all members of each clan by their unique spot patterns, assigned birthdates (± 7 days) to natal animals based on cub appearance when first seen [42], and assigned a sex to each individual based on the shape of the glans of its erect phallus [43].

We obtained recordings of whoop vocalizations in two ways (electronic supplementary material, figure S2). First, from April 2010 to January 2011 and from July 2014 to April 2016, observers deployed a hand-held directional microphone and digital recorder from the windows of off-roading vehicles used as mobile blinds. Second, custom-made sound-, movement- and position-recording collars were deployed from January to March 2017 on five adult females from the Talek West clan. Recording periods and methods are indicated for each whoop bout in the electronic supplementary material, table S1.

**Figure 1.**
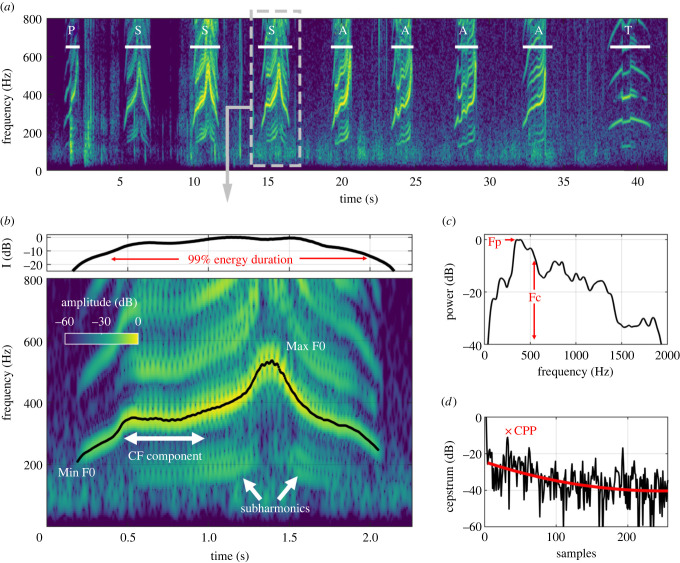
Acoustic analysis pipeline. (*a*) Spectrogram of a whoop bout (resolution 17.1 ms × 3 Hz, 90 dB dynamic range). Whoops within each bout were manually isolated and classified as either ‘S’ (symmetric), ‘A’ (asymmetric),‘T’ (terminal), or ‘P’ (preliminary) whoop category. (*b*) Spectrogram of a single (S type) whoop. For each whoop, the fundamental contour was manually traced, and a variety of call features were extracted, including fundamental frequency parameters, duration of the ‘CF’ or ‘constant-frequency’ portion of the whoop, and duration of subharmonics; (*c,d*) illustrate call features acquired from spectral and cepstral analyses, respectively. See Methods and the electronic supplementary material for information on call parameters. (Online version in colour.)

We isolated whoop bouts from both types of digital field recordings, noted the time, date and identity of the calling hyena and matched this information with the age, sex and clan membership of the caller. We then cut each whoop bout into single whoops for analysis (figure 1), using only whoops from adult hyenas that were at least 24 months old, and thus reproductively mature [44], to eliminate the possibility that young hyenas might not yet have learned a potential group signature.

We classified whoops into four types (figure 1*a*) based on a classification scheme modified from East & Hofer [41]. Preliminary whoops (P type) are often emitted at the beginning of the whoop bout and are typically very short relative to other whoops in the bout. Symmetric (S type) whoops resemble a flattened bell curve, with the peak frequency near the centre of the call. By contrast, asymmetric (A type) whoops have a long constant-frequency (CF) portion that rises to peak frequency toward the end of the call. Terminal (T type) whoops are often the last whoop in a bout. They maintain a relatively constant, low frequency and are often of lower amplitude than the other whoops in the bout [38].

### (b) Acoustic processing and feature extraction

Recordings were resampled to a common sample rate of 32 kHz. Each signal was then processed individually using custom-written software in Matlab 2019a [45] to extract a range of acoustic parameters with focus on features that were robust to long-range transmission (table 1; see the electronic supplementary material for full details).

**Table 1.** Acoustic features are extracted from each whoop.

abbreviation	measurement	units
dur	duration of call (99% energy criterion)	[seconds]
dur.cf	duration of CF component	[seconds]
dur. upsweep	duration of upsweep (until max frequency)	[seconds]
dur. subharm	duration of call with dominant subharmonics (energy > harmonics)	[seconds]
endtime.cf	end time of CF component relative to call	[fraction of call]
endtime.upsweep	end time of upsweep (max frequency) relative to call	[fraction of call]
freq. centroid	centroid frequency	[kHz]
freq. peak	peak frequency	[kHz]
freq. min	min fundamental frequency	[kHz]
freq. max	max fundamental frequency	[kHz]
freq. mean. cf	mean fundamental frequency within CF component	[kHz]
harmonic. ratio. total	harmonic to subharmonic energy ratio within entire call	[dB]
harmonic. ratio. cf	harmonic to subharmonic energy ratio within CF component	[dB]
mean.entropy	mean spectral entropy within 99% energy duration	[0(pure tone) to 1(white noise)]
cpp.mean	mean cepstral peak prominence	
cpp.sd	standard deviation of cepstral peak prominence across time slices	

First, the 99% energy duration was extracted, and within this window, the peak frequency and centroid frequency were estimated [46]. Then, the fundamental frequency contour was manually traced by the analyst (figure 1*b*), allowing for estimation of the minimum and maximum contour frequencies. Three parts of the signal were then estimated from the contour: the initial CF portion of the whoop (defined as the period where the contour was within ± 10% of the median frequency of the contour prior to the peak contour frequency); the upsweep portion of the whoop (from end of CF component to peak frequency); and any periods with significant subharmonics (where the total energy in the subharmonics exceeded energy in fundamental frequency and harmonics). Features extracted from these periods included the total duration of each component, and the relative time point of the end of the CF portion and the end of the upsweep.

Finally, the signal was resampled to 8 kHz and divided up into 4 ms blocks with 3.5 ms overlap. For each block, the continuous spectral entropy [47] and the cepstral peak prominence (CPP) [48] was calculated, and the mean was taken across the total 99% energy duration.

### (c) Using random forests to predict clan membership and individual identity

To test whether whoops contain clan and/or individual signatures, we trained random forest classifiers [49] to predict either clan or individual identity based on the set of extracted acoustic features. Random forest classification is a supervised machine learning algorithm that uses a set of decision trees (i.e. a ‘forest’) to classify objects that are represented by measured features of the objects. Each tree in a forest attempts to parsimoniously split the training objects into the correct categories based on a random subset of object features, and a majority rule is used to produce a final ensemble classification across trees.

#### (i) Testing the clan signature hypothesis

We first tested whether spotted hyenas use generalizable acoustic features that help differentiate clan identity from whoops irrespective of individual identity. To investigate this, we split our full dataset (*n* = 514 whoops from 39 hyenas in four clans; electronic supplementary material, table S1) into training and test datasets. The training dataset consisted of whoops from all except one randomly selected hyena from each clan. These remaining whoops were used as the test dataset (electronic supplementary material, figure S3a). This cross-validation ensured that features had to generalize across hyenas and that the random forest classifier could not learn to recognize clans through recognizing individuals. We then trained a random forest classifier with 500 decision trees with the number of nodes set to the size of the training set and measured the classification accuracy as the number of correctly identified whoops in the test dataset. This resulted in the whoops of any single hyena being in either the test or training dataset, but not both, thus preventing the classifier from learning the characteristics of individual hyenas and ensuring that accuracy only reflects features that generalize across hyenas within a clan. To assess the accuracy of predictions, we repeated this process 1000 times, with a random hyena from each clan selected for the test data each time.

Because animals varied in their number of recorded whoops, each random selection of test individuals resulted in a different proportion of correctly assigned whoops owing to chance. As a null model, we therefore calculated a weighted expectation (WE), which is the expected proportion correct owing to chance alone.

Because most male hyenas disperse from their natal clans [31,50], males may retain their natal group signature instead of learning the vocal signature of the clan in which we recorded them. We tested for this possibility by rerunning separate analyses with males only or with females only.

#### (ii) Testing the individual signature hypothesis

To prevent the random forest from assigning individual identity based on autocorrelated variation present within a whoop bout instead of common variation among an individual's whoop bouts, we held out one bout from each individual for the test dataset and used the remaining whoops as training data (electronic supplementary material, figure S3b). This required reducing the dataset to all hyenas having two or more whoop bouts with at least three whoops (*n* = 312 whoops from 13 hyenas, 9–54 whoops per hyena; electronic supplementary material, table S1). As before, we then trained a random forest classifier with 500 trees to predict individual identity on the training dataset and measured performance as the fraction of whoops in the test dataset with correctly assigned individual identity. We repeated this procedure 1000 times, each time withholding a randomly selected whoop bout from each individual for the test dataset. As above, a WE probability was calculated as the fraction of whoops that would be correctly assigned to the individual by chance.

To test the possibility that individual classification accuracy was influenced by recording method, we ran this analysis separately on data from microphone recordings and collar recordings. We also reran the random forest analysis with males and females separated to determine whether one sex has more individually distinctive whoops than the other.

Because whoop type affected the accuracy of assignment, we created a final dataset of only A and S type whoops and retested both clan and individual signature hypotheses (see figures for sample sizes and the electronic supplementary material, table S1 for full details on all datasets). We constructed confusion matrices for all random forest analyses (electronic supplementary material, figures S4 and S5). Finally, we calculated importance as the mean decrease in individual classification accuracy when each feature is excluded from the classification model using the ‘importance’ function from the randomForest package (table 1).

#### (iii) Testing whether signal redundancy improves caller identification

Finally, we investigated how the sequential nature of natural whoop bouts influences classification accuracy and thus might help alleviate uncertainty about caller identity. To do this, we simulated a receiver's likelihood of assigning a whoop bout to the correct caller based on multiple whoops in the bout. Within a random forest model, we calculated each test bout's accuracy by calculating the proportion of decision trees that classified each bout to each of the hyenas in the dataset given one whoop, two whoops, etc. This gave us a ‘probability’ that each bout belongs to each hyena for each number of whoops within the bout.

We then calculated the average correct probability across all the random forest models to account for variations in prediction accuracy from using different whoops for training and testing. This analysis was only conducted when models reached an average accuracy above random guess because we would not expect redundancy to meaningfully increase accuracy in such cases. We used the random forests trained with only ‘A’ and ‘S’ whoops but otherwise maintained the natural order of whoops within the bout.

All analyses and figures were generated in RStudio with R v. 4.2.0 (22 April 2022) [51] and Bookdown 0.26 [52]. We analysed data using the tidyR 1.2.0 [53] and randomForest 4.7.1.1 [54] packages, and created figures using gplots 3.1.3 [55], ggplot2 3.3.6 [56] and cowplot 1.1.1 [57]. Diagrams were created in Powerpoint and colours were generated from viridis 0.6.2 [58].

## 3. Results

**Figure 2.**
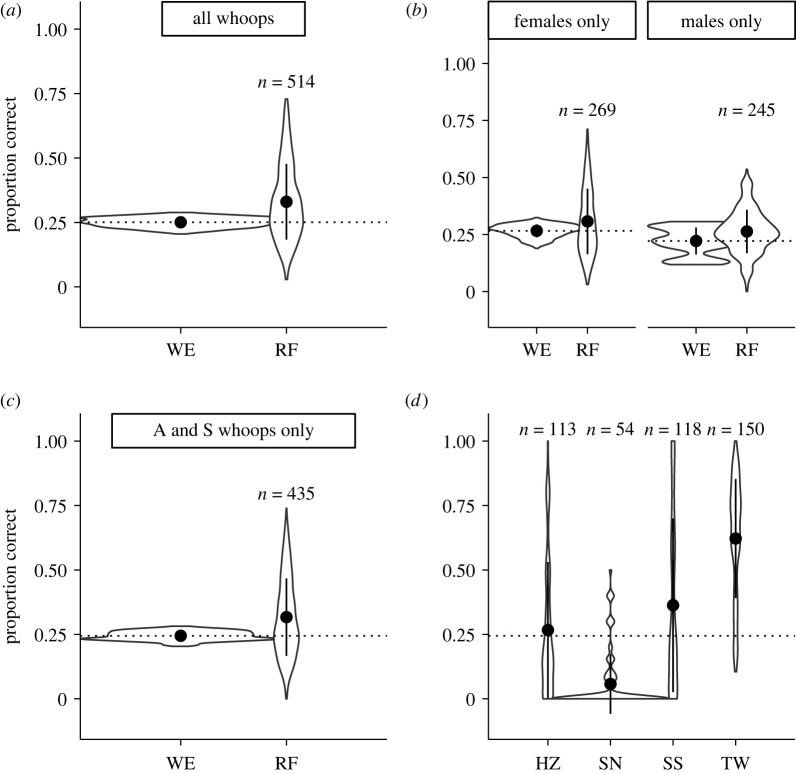
Lack of clan signatures in single whoops. Violin plots of proportion of test data correctly assigned to clan from 1000 random forests (RFs). (*a*) Comparing random WE to performance of the RF, using all whoop types. (*b*) Comparing WE and RF for datasets composed of whoops from females only and males only. (*c*) Comparing WE and RF using only ‘A’ and ‘S’ type whoops. (*d*) Random forest accuracy of test data for each hyena clan, using only ‘A’ and ‘S’ type whoops. Points and bars represent means and standard deviations. of random forest accuracy. Dotted line indicates mean random WE for that dataset.

The random forest model for assigning clan membership was no more accurate than expected by chance (figure 2; mean: 0.32, s.d.: 0.15, chance: 0.24), and neither sex of the calling animals (figure 2*b*) nor whoop type (figure 2*c*) had any influence on clan identification.

**Figure 3.**
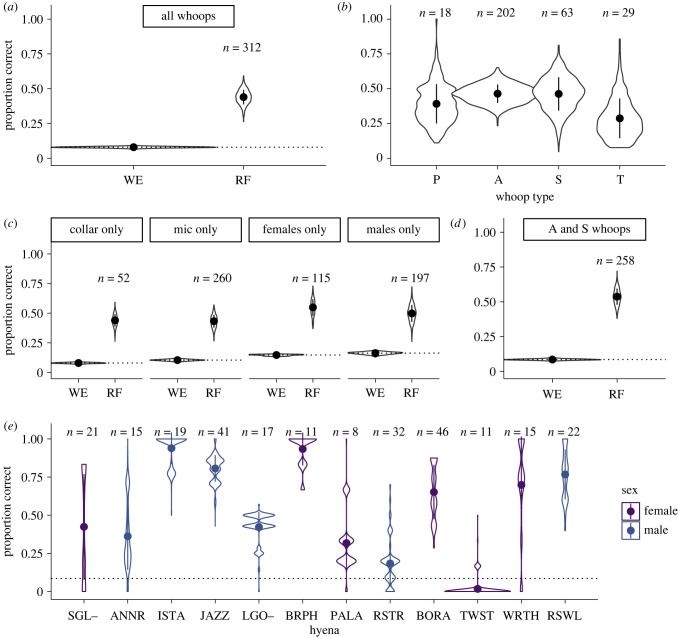
Individual signatures in single whoops. Violin plots of the proportion of test data correctly assigned to individual from 1000 random forests (RFs). (*a*) Comparing random WE to performance of the RF, using all whoop types. (*b*) Random forest accuracy by whoop type. (*c*) Comparing WE and RF for datasets composed of collar recordings only, microphone recordings only, females only and males only. (*d*) Comparing WE and RF using only ‘A’ and ‘S’ type whoops. (*e*) Random forest accuracy for individual hyenas, using only ‘A’ and ‘S’ type whoops. Points and bars represent means and standard deviation of random forest accuracy. Dotted line indicates mean random WE for that dataset. (Online version in colour.)

By contrast, the random forest model for assigning individual identity was much more accurate than expected by chance (figure 3; mean: 0.54, s.d.: 0.058, chance: 0.09). Again, these results held true regardless of sex or recording method, even with the reduced sample sizes in these datasets (figure 3*c*).

The accuracy of individual assignment varied with whoop type (with ‘A’, ‘S’ and ‘P’ assigned more accurately than ‘T’; figure 3*b*) and final analyses were conducted with only ‘A’ and ‘S’ type whoops (figures 2*c* and 3*c*). The accuracy of assignment to clan and individual varied considerably among the clans (figure 2*d*) and individual callers (figure 3*e*). Further, clans that bordered each other were not more likely to be confused (electronic supplementary material, figure S4) and individuals within a clan were not more likely to be confused with one another than with individuals from different clans (electronic supplementary material, figure S5).

**Figure 4.**
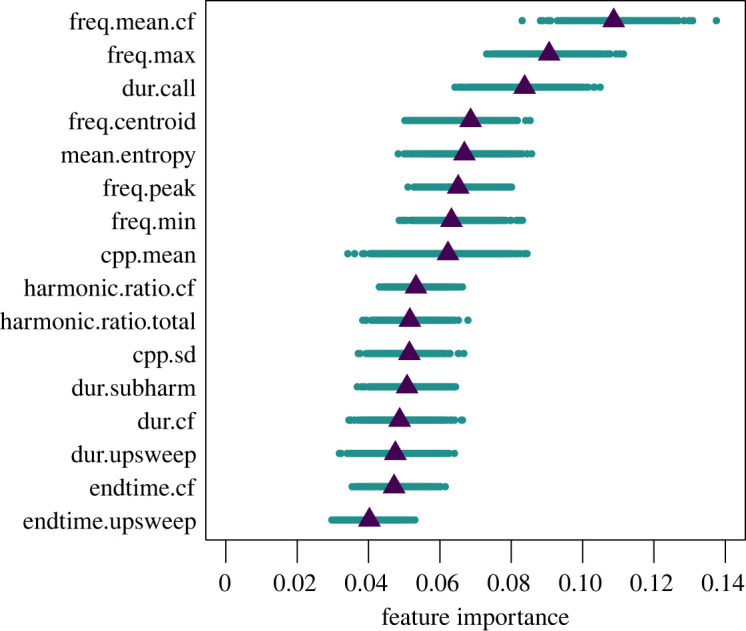
The importance of each whoop feature in predicting individual identity. Feature importance is measured as the mean decrease in accuracy when that feature is removed from the random forest analysis. See table 1 for full feature names and descriptions. (Online version in colour.)

Some call features were more important than others for correctly predicting individual caller identity (figure 4). The top features were the mean frequency of the CF portion of the whoop, the maximum frequency and the call duration.

**Figure 5.**
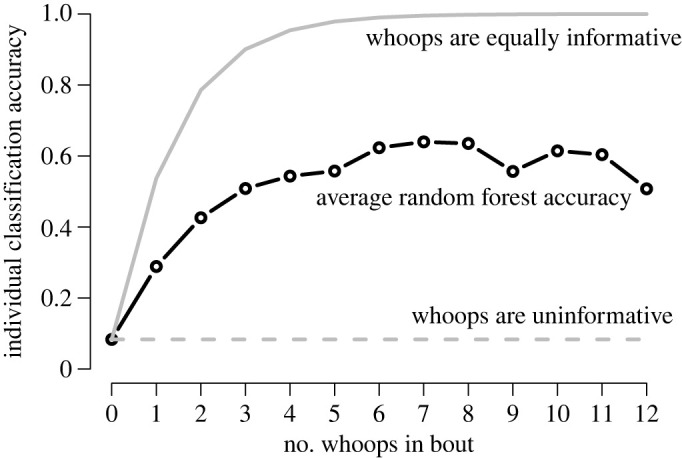
The sequential nature of whoop bouts increases assignment accuracy. The expected proportion of correct guesses of caller identity improves with number of whoops examined in the bout based on the random forest predictions (black line with points). The average accuracy as a function of the number of whoops in a bout given the null hypothesis that whoops are uninformative (dashed grey line) and given that each whoop is equally informative (using the average accuracy of the 1000 random forests trained with ‘A’ and ‘S’ type whoops, 54%: solid grey line).

Our analysis of whoop redundancy within a bout supported the hypothesis that the repetitive nature of the whoop bout increases receiver certainty about the identity of the caller. With more whoops in a bout, the proportion of correct guesses increases, although not at the rate expected if all whoops are equally informative (figure 5).

## 4. Discussion

### (a) Individual but not group signatures

Theory predicts that species will evolve signals that meet their minimum needs [3] while using as few categories of signals as possible to maximize detection and discrimination [1]. Given the size [25,26], dynamic membership, and spatial dispersion of hyena groups (i.e. neighbouring clans hear each other's whoops, making ‘familiar or not’ discrimination insufficient), they are a strong candidate species for a group signature. This group signature would allow hyenas to categorize callers as ‘clan-mate or not’, thereby facilitating the coordination and recruitment of clan-mates and detection of territory intruders. We found multiple call features that facilitate individual discrimination, but no evidence of a group-level signature. This suggests several possibilities about the relationship between hyenas' fitness-critical needs and the resultant structure of their communication: (i) recognition of large numbers of group-mates by voice alone may not be as costly as expected; (ii) group signatures may be more costly than expected; and (iii) group signatures may not meet hyenas’ minimum needs, nor any need beyond those already met by individual signatures.

There is some evidence that vocal recognition of many individuals is not costly enough to require the categorical reduction that group signatures would provide. Although it is difficult to determine experimentally the number of voices recognized by humans, we do know that humans can accurately distinguish many individuals from voice alone [59–62]. In addition, several studies in non-human animals have demonstrated individual vocal recognition [63–68] (but see [69]). Some species are clearly capable of recognizing numerous individual callers (approximately 100 individuals in African elephants [70]) and even associate callers with traits lying on multiple axes (such as rank and kinship [71,72]). Hyenas clearly have the cognitive capacity to recognize and remember clan-mates as individuals [28,30,73]; perhaps the development of a group signature is more costly than the memory required to recognize 125 + voices.

Evolving a group signature would also require concurrent evolution of flexible vocal production learning [74], a trait that is argued to be relatively rare in animals [75]. Without this trait, an individual would be unable to learn and produce a new group signature after changing groups. During clan fission [76] and male immigration [31,50], hyenas would need to learn how to produce the group signature of their new clan. Although hyenas might be capable of flexible vocal production, our results suggest that a group signature is not the impetus. Instead, the individual signatures we detected would rely on hyenas' associative learning and flexible vocal comprehension [75]. Male hyenas especially may have to learn to recognize the individually distinct calls of an entirely new suite of group-mates while other animals in a male's new clan must learn to recognize the new immigrant's voice.

The lack of group signatures in whoops also suggests that a simple ‘group-mate or not’ classification either is unnecessary given the presence of individual signatures or is insufficient for spotted hyenas to respond adaptively to these vocalizations. This is consistent with the hypothesis [6] that individual signatures in vocal calls are tied to the evolution of differentiated social relationships in complex societies. Hyena society does exhibit characteristics that support the evolution of identity signalling, specifically, large group size, complex and repeated social interactions with both kin and non-kin, dominance hierarchies, and territoriality [4]. Spotted hyenas show social preferences for certain group-mates based on kinship and dominance [77,78], and social alliances can restructure the social hierarchy [79] to influence rank and fitness [80]. Therefore, long-distance calls encoding individual identity may be crucial to the functioning of hyena societies, allowing group members to manage numerous social relationships occurring over large spatial scales. The memorization of these individual signatures then provides the requisite group membership information for mediating an effective cooperative territory defence, thus obviating the need for a group signature.

Interestingly, some whoop types and some individuals were more difficult than others to categorize correctly; however, we cannot disentangle whether this is a product of our dataset or whether instead it represents meaningful differences among whoop types (as in lion roar types [81]) and individual voices. ‘A’ type whoops may be more easily classified because they are over-represented in the dataset or ‘A’ whoops may be more common within whoop bouts because their protracted CF portion is a good indicator of individual identity (figure 4). T whoops may have been poorly classified owing to their under-representation, or they may not encode individual identity at all. The variation in individual assignment accuracy may be an artefact of the recordings we happened to obtain (e.g. our two recorded bouts happen to span the variation produced by that individual), or it might reflect real challenges that hyenas face in the wild. Some individuals (e.g. low ranking individuals) may benefit from being more difficult to identify, or the acoustic space may not be large enough to accommodate a large number of distinct signatures.

### (b) Call features adapted for long-range transmission

To solve the challenge of communicating individual identity across expansive territories, evidently spotted hyenas have evolved to encode identity information in features that are particularly robust to long-range propagation. The call features that were most important for discriminating individuals included a number of frequency measures (mean frequency of the CF portion of the whoop, maximum and minimum frequency of the fundamental, and centroid frequency), call duration and measures of noisiness (entropy) and dysphonia (CPP mean). It is important to note that hyenas may rank these features differently or use additional call features not identified in our study. However, frequency features are commonly involved in individual vocal recognition [82] and other species that rely on long-range signalling appear to exhibit similar adaptations, including wolves [83], lions [81] and bottlenose dolphins [7]. The importance of the entropy and CPP measures suggests that hyenas might also attend to the biphonic components, which are common in some hyena whoops. While these features are unlikely to transmit over long distances, they are a common identifier in the voices of several species [82].

In addition to identifying individual hyenas, frequency features may also make it easier for hyenas to locate a whooping individual [38] as calls with a wide frequency range are expected to facilitate localization of the sound source [84]. Low frequencies are also easier to locate in most situations [20] and are thus advantageous for long-distance calls that advertise the caller's location. The high-frequency portions, which degrade more quickly, may allow a receiver to ascertain the distance of the caller from it while the low-frequency portions of the call ensure it reaches as many receivers as possible.

### (c) The value of repetition

Signal redundancy has been shown to improve recognition accuracy in evolutionary agent-based models of recognition [85], so redundancy within whoop bouts probably increases both the probability of detection and the receiver's ability to identify the caller. This notion was supported by our calculations of increasing classification accuracies over the course of whoop bouts, although this increase did not reach the classification accuracies expected if each whoop was equally informative and accuracy with each additional whoop followed a Bayesian updating rule. This reduced accuracy is probably owing to each whoop within a bout *not* being an independent observation. Each random forest was trained on one bout fewer than the number of bouts available for each individual. This ensured that random forest accuracy was owing to individual-level, and not bout-level, characteristics, but also resulted in some bouts with low accuracy. This correlated error is certainly an artefact of our machine learning approach, but may also reflect real challenges experienced by receivers in the wild when consecutive signals have redundant information leading to correlated errors.

In systems where signallers are unable to predict how signals degrade during transmission or the amount of noise that their receivers will experience, additional repetition increases the chance that a signal will be detected and correctly decoded. When combined with our results regarding feature importance, this repetition becomes more powerful. Mean fundamental frequency of the first whoop may allow a hyena to narrow the potential caller set (e.g. to ‘adult females’), while each successive whoop provides opportunity to closely attend to other acoustic features and further narrow the potential identify of the caller. It is important to note that our calculation of the prior probability that a bout belongs to any individual will be much different than the prior probabilities that a hyena will encounter in the wild. Although most spotted hyenas must discriminate between many more than the 12 individuals we distinguish here, they probably also have prior information regarding which individuals are nearby or in a particular direction. The repetition of whoops within the bout provides multiple opportunities for receivers to localize the caller [86] while also deriving information from tonic features of the bout, specifically the inter-whoop-interval [36].

This serial redundancy within whoop bouts also allows for subsequent divergence between repeated elements and co-option of a derived element for a new purpose [24]. For example, whoop bouts often start with a ‘P’ type, truncated whoop, a simple tonal call that may serve as an alerting component [87]. Thus, it is possible that each whoop type conveys a different kind of information, that the sequence conveys information, or even that two hyenas share similar ‘S’ whoops and different ‘A’ whoops. Unfortunately, our sample size was not large enough to directly test these hypotheses here.

Although there have been a number of studies on increased redundancy in calls owing to increased noise in the environment, to our knowledge, no studies have previously attempted to quantify the increase in accuracy of information transfer as the redundancy of the signal increases. There is an important push in the animal behaviour literature to investigate degenerate signals in multi-modal signalling systems [23,88], especially when studying the interaction between social and communicative complexity [89]. We suggest this should also extend to redundancy over time because animals are constantly integrating signals and new information into their decisions.

### (d) The function of advertisement whoops

It is noteworthy that, although whoops are used to recruit clan members for collective action, a large proportion (47.1% [37] to 60% [30]) of whoop bouts are ‘spontaneous’ or ‘slow’ and do not appear to recruit individuals [36], suggesting they serve an additional function. We concur with East & Hofer's [38] suggestion that spontaneous whoops display the identity and location of the caller, and as such they can help hyenas keep track of conspecifics and thus simplify the task of discriminating between conspecifics from long-range degraded signals by informing the prior probability of where conspecifics should be located. However, we also suggest that these bouts may reinforce the templates, or mental representations of calls, of receivers within hearing distance [1,90]. This function of spontaneous whoops may be especially important given that receivers must discriminate among many group-mates and also between group and non-group-mates with the potential for correlated error and without the benefit of a group signature. Such memory reinforcement should improve future detection and discrimination as it does in humans [1].

## Data Availability

All data and code will be publicly available online at: https://doi.org/10.5061/dryad.djh9w0w2h [91] and https://github.com/kdslehmann/HyenaWhoopSignatures.git. The data are provided in the electronic supplementary material [92].
